# Economic evaluations of non-communicable diseases conducted in Sub-Saharan Africa: a critical review of data sources

**DOI:** 10.1186/s12962-023-00471-7

**Published:** 2023-08-28

**Authors:** Samantha A Hollingworth, Glory-Anne Leaupepe, Justice Nonvignon, Ama Pokuaa Fenny, Emmanuel A. Odame, Francis Ruiz

**Affiliations:** 1 School of Pharmacy, University of Queensland, Brisbane, Australia; 2 School of Public Health, University of Ghana, Accra, Ghana; 3 Institute of Social, Statistical and Economic Research, University of Ghana, Accra, Ghana; 4 Dept of Medical Affairs, Korle Bu Teaching Hospital, Accra, Ghana; 5 Department of Global Health and Development, London School of Hygiene and Tropical Medicine, London, UK

**Keywords:** Sub-Saharan Africa, Non-communicable diseases, Economic evaluations, Costs, Data sources

## Abstract

**Background:**

Policymakers in sub-Saharan Africa (SSA) face challenging decisions regarding the allocation of health resources. Economic evaluations can help decision makers to determine which health interventions should be funded and or included in their benefits package. A major problem is whether the evaluations incorporated data from sources that are reliable and relevant to the country of interest. We aimed to review the quality of the data sources used in all published economic evaluations for cardiovascular disease and diabetes in SSA.

**Methods:**

We systematically searched selected databases for all published economic evaluations for CVD and diabetes in SSA. We modified a hierarchy of data sources and used a reference case to measure the adherence to reporting and methodological characteristics, and descriptively analysed author statements.

**Results:**

From 7,297 articles retrieved from the search, we selected 35 for study inclusion. Most were modelled evaluations and almost all focused on pharmacological interventions. The studies adhered to the reporting standards but were less adherent to the methodological standards. The quality of data sources varied. The quality level of evidence in the data domains of resource use and costs were generally considered of high quality, with studies often sourcing information from reliable databases within the same jurisdiction. The authors of most studies referred to data sources in the discussion section of the publications highlighting the challenges of obtaining good quality and locally relevant data.

**Conclusions:**

The data sources in some domains are considered high quality but there remains a need to make substantial improvements in the methodological adherence and overall quality of data sources to provide evidence that is sufficiently robust to support decision making in SSA within the context of UHC and health benefits plans. Many SSA governments will need to strengthen and build their capacity to conduct economic evaluations of interventions and health technology assessment for improved priority setting. This capacity building includes enhancing local infrastructures for routine data production and management. If many of the policy makers are using economic evaluations to guide resource allocation, it is imperative that the evidence used is of the feasibly highest quality.

**Supplementary Information:**

The online version contains supplementary material available at 10.1186/s12962-023-00471-7.

## Introduction

Sub-Saharan Africa (SSA) has a high burden of non-communicable diseases (NCD) which include cardiovascular disease (CVD), diabetes, cancer, respiratory disease, and mental health conditions [1]. Despite a population of over one billion people, less than 1% of the world’s financial resources for health are spent in SSA; it has only 3% of the global health workforce, while having 24% of the global burden of disease [2]. The rising impact of NCDs in Africa have led to predictions that associated deaths would exceed those linked to maternal, perinatal, nutritional, and communicable diseases combined by 2030 [3].

Many African countries are seeking to achieve Universal Health Coverage (UHC), one of the Sustainable Development Goals (SDG), as part of ambitions to improve access to health services for their citizens [4]. However, due to economic challenges linked to a changing aid environment, a fragmented and inefficient structure of domestic and international health financing, and a lack of regulation or oversight of the private health sector, notwithstanding also the ongoing impact of the COVID-19 pandemic on health systems, achieving meaningful UHC will likely be difficult [5, 6].

Achieving UHC will in part depend on ensuring that available resources are used to maximise health benefits where possible. This can be achieved by building sustainable and locally relevant evidence-informed priority-setting systems utilising approaches such as health technology assessment (HTA) [7]. HTA aims to synthesise evidence from several disciplines to inform policy and clinical decision making around the introduction of health technologies, such as medicines, devices, and diagnostic approaches. It is a globally accepted approach for bringing together evidence on costs and clinical effectiveness, whilst also considering broader social values including equity, and is usually embedded in a well-defined multi-stakeholder process [8]. The benefits of HTA have been demonstrated in many high and upper-middle income countries by informing resource allocation decisions [9].

There are many aspects to consider when establishing HTA systems, but a core input is the availability of locally relevant data and evidence; this is especially challenging in the absence of strong health information systems [10]. For effective HTA, there is a need for high-quality data covering a number of key informational domains: epidemiology (such as prevalence and incidence of disease), clinical effectiveness, health outcomes (such as health related quality of life), resource use and costs, and equity [11]. Many SSA countries lack comprehensive and robust locally-generated data [12]. They may not have guidelines for undertaking economic evaluations within an established HTA system nor adequate capacity to conduct and assess relevant HTA studies [13]. Also lacking in most African countries are independent institutions or institutional processes to conduct HTA assessments [8]. Despite these challenges, economic evaluations focused on African settings have been undertaken, with many of these in relation to communicable disease. While fewer in number, there are published economic evaluations of NCD-related interventions in African settings but there are some concerns about their quality [14]. This has highlighted questions regarding the underpinning data sources informing key parameters in the analyses.

## Methods

We aimed to explore the sources and quality of data used in economic evaluations of interventions to prevent or treat CVD and diabetes in SSA since 2000. Furthermore, we examined the content, assessed the reporting and methodological standards, and ranked the quality of evidence of the included studies.

### Literature search

We conducted a systematic literature search to identify economic evaluations pertaining to SSA. We searched PubMed, Embase, Scopus, and CINAHL from 1 January 2000 to 14 August 2021. The main search terms were economic evaluations, costs, Sub-Saharan Africa, and non-communicable diseases. The studies were screened in two stages – firstly the title and abstract, and secondly the full text. We excluded evaluations published before 2000, those concerning communicable diseases, not pertaining to SSA, or not in English. We only included articles that were full economic evaluations (i.e. with both costs and outcomes of two or more interventions), peer reviewed, and in cardiovascular disease or diabetes.

### Evaluation of data sources

We developed an extraction template to record four aspects: (a) general study characteristics; (b) methodological and reporting characteristics relative to an economic evaluation Reference Case developed by international experts [15]; (c) quality of data sources covering the six data domains of HTA [11, 16]; and (d) author comments on data sources and quality. Firstly, the study characteristics included general information such as the first author, institution of first author, journal, type of economic evaluation, study perspective, source of funding, discount rate, time horizon, type of model (empirical [trial-based] or model), currency, and the type of sensitivity analysis used [17, 18]. Secondly, we used the International Decision Support Initiative (iDSI) reference case [15, 19, 20] to create a checklist of 40 questions for methodological and reporting standards. Each question was assigned a 1 (yes), 0 (no or unclear). Thirdly, we ranked the quality of data sources used for six domains of data - clinical effectiveness, costs, epidemiology, quality of life (outcomes), resource use, and equity [11] - adapted from a hierarchy of evidence [16]. We ranked each individual data source within the six domains for each of the studies (where applicable) where the rankings ranged from one to six levels. For ranking of effectiveness, there may be two levels within a rank to distinguish between evidence from a meta-analysis of trials (denoted by +) and a single trial [16]. We modified the ranked order of two domains (resources and service use, and costs) to better reflect the higher ranking of expert opinion in Sub-Saharan Africa given the sparsity of data. For some domains – especially epidemiology, resource use and costs - multiple data sources were included within a data domain. We calculated the proportion of sources in each of three levels of evidence – high (ranks 1 and 2), medium (rank 3), and low (ranks 4 to 6). Lastly, we extracted information from the Discussion section of each study to examine any comments from the authors about data sources and quality [8]. We descriptively analysed the main themes from those papers that discussed the issues. We used Microsoft Excel to record data.

## Results

### Search results

**Fig. 1:**
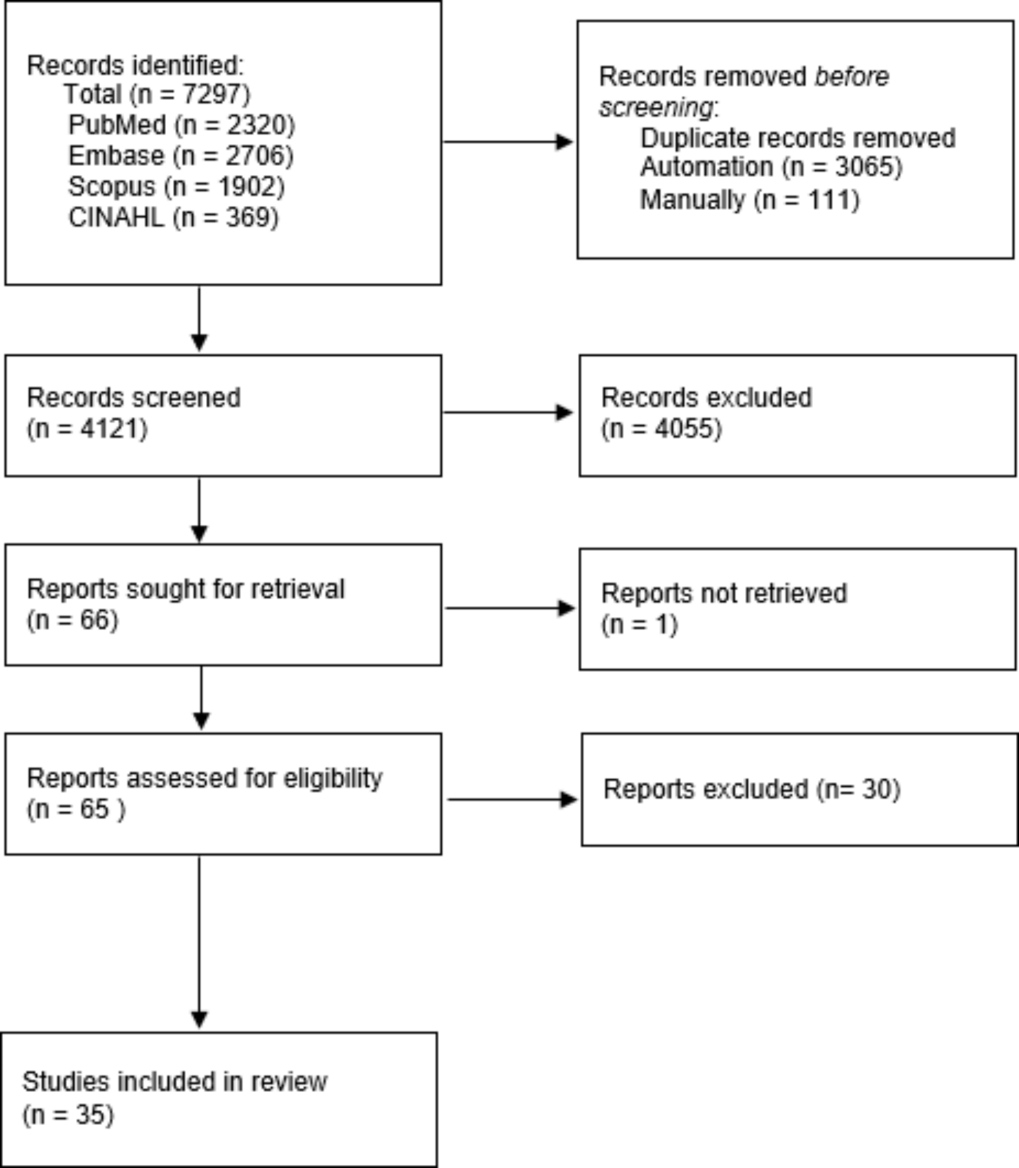
PRISMA flow chart

The systematic search yielded 7,297 articles but after removing duplicates there were 4,121 studies remaining (Fig. 1). Screening of titles and abstracts led to 65 full text reviews; ultimately 35 evaluations were selected [17, 21–54].

### General characteristics of studies

The evaluations were published between 2000 and 2021 (Table 1, Additional File 1) and most were exclusively concerned with SSA settings, except for two: one study included South-East Asia, and another included all LMICs (only data for SSA were extracted). There were 28 (80%) cost-effectiveness analyses (CEA) where non-monetary measures of health outcomes were used, two cost-consequence analyses (6%), and four (11%) cost benefit analyses. One study modelled the impact of a tax on sugar sweetened beverages in Zambia on deaths averted, life years gained, and revenues generated [36]. The outcomes measures in the studies included: DALYs (n = 14, 40%), QALYs (n = 6, 17%), and currency (n = 4, 11%; further information on other outcomes is available in Additional File 1). Most evaluations were models (71%), the rest were empirical. Markov was the most common model type (n = 13) followed by retrospective (n = 5) and microsimulation (n = 4). The perspective of the evaluations was predominantly from the healthcare sector (n = 21, 60%), followed by society (n = 7, 20%). The interventions in the evaluations were targeted at cardiovascular disease (n = 13, 37%), non-communicable disease generally (n = 8, 23%), hypertension (n = 7, 20%), and diabetes (n = 7, 20%).

**Table 1:** Characteristics of selected studies with majority category noted (N = 35)

Aspect	n	%
Single country in Africa	29	83
First author, affiliation is an African institution	18	51
First author, affiliation is an academic institution	34	97
First author, second affiliation has been given	4	11
Corresponding author, affiliation is an African institution	15	43
Corresponding author, affiliation is an academic institution	30	86
Intervention - population target ^a^	25	71
Intervention type (management, majority) ^b^	16	46
Intervention measure – DALY	14	40
Intervention measure - any utility-based	24	69
Evaluation type - CEA only	11	31
Evaluation type - CEA or CUA	28	80
Evaluation design – modelling	25	71
Model type - Markov	13	37
Time horizon (years)	Median	10
Perspective – health care system	21	60
Discount rate (3%)	21	60
Uncertainty - sensitivity analysis: univariate	14	40
Uncertainty - sensitivity analysis: any type	23	66
Currency – United States Dollars	20	57
Currency – Local	21	60
Currency – year	Median	2014
Threshold - GDP or GNI based	20	57

a population or individual; b medicine, management, population program, or policy

CEA cost effectiveness analysis, CUA cost utility analysis, DALY disability adjusted life year, GDP Gross domestic product, GNI gross national income

### Adherence to iDSI reference case reporting standards

All the evaluations (100%) stated their intervention, outcome, and parameter sources (Table 2). Most evaluations stated the population of interest and cited parameter sources (97%), general limitations (94%), the comparator [transparency] (91%) and time horizon (91%). Most evaluations (> 80%) included a conflict-of-interest statement, funding source, clearly stated the comparator [comparators], stated the costs in local and US dollars, and stated the perspective. The weaker elements included providing budget impact estimates (54%), considerations of equity (51%), and subgroup analysis (46%, Table 2).

Three out of four evaluations indicated the use of a discount rate, and 22 studies applied a 3% discount rate for both inputs and outputs (some studies used more than one discount rate). Three of the selected studies used discount rates in sensitivity analysis that were greater than 3%; two studies applied a discount rate of 10% [22, 54]. For those six studies that did not apply discounting, in only two the stated time horizon of the analysis was short (1 year). Four in five evaluations stated a time horizon.

**Table 2:** Reporting standards (adapted from iDSI reference case)

Reporting Standards	n	% (N = 35)
Population stated	34	97
Intervention stated	35	100
Comparator stated	32	91
Outcome stated	35	100
Limitations stated (general)	33	94
Conflict of interest statement included	30	86
Funding source stated	29	83
Comparator clearly stated	31	89
Reported incremental cost effectiveness ratio (ICER)	24	69
Parameter sources stated	35	100
Parameter sources cited	34	97
Weighting methods stated	24	69
Costs in local currency	30	86
Costs in United States Dollars	29	83
Time horizon clearly stated	32	91
Discounting for both costs and outcomes clearly stated	25	71
Perspective clearly stated	29	83
Subgroup analysis performed/stated	16	46
Reported results of sensitivity analysis	25	71
Impact on budget stated	19	54
Influence of equity considerations stated in the paper	18	51
**Average Score**	**17.1**	**81**

### Adherence to iDSI reference case methodological standards

All the economic evaluations characterised the decision problem (100%), and all but one characterised the limitations and reported costs that were consistent with the perspective (97%, Table 3). Many studies (> 80%) included a declaration of interest statement, used the standard of care as the comparator, used a limited societal perspective, and reported direct health costs.

Fewer than half of the studies (< 50%) stated whether a lifetime horizon was used, performed a sensitivity analysis of parameter sources, included a budget impact assessment, or used a systematic review to identify relevant evidence. In terms of exploring uncertainty in the economic evaluation, 19 studies (54%) undertook sensitivity analysis on model structure. In terms of parameter uncertainty, 16 studies (46%) applied deterministic sensitivity analysis and 18 studies (51%) undertook probabilistic sensitivity analyses; nine studies conducted both (Table 3).

**Table 3:** Methodological standards (adapted from iDSI reference case)

Methodological Standards	n	% (N = 35)
Decision problem characterized	35	100
Limitations characterized	34	97
Declaration of interest reported	30	86
Comparator is standard of care	29	83
Systematic review used	12	34
DALYs as main outcome	21	60
Costs are relevant to reported perspective	34	97
Costs include implementation	20	57
Lifetime time horizon used	16	46
3% discount rate used	21	60
Discount rate used for costs and effects	23	66
Limited societal perspective used	28	80
Direct health costs reported	30	86
Subgroup analysis performed/stated	19	54
Structural sensitivity analysis performed	19	54
Sensitivity analysis of parameter source performed (deterministic)	16	46
Sensitivity analysis of parameter precision performed (probabilistic)	18	51
Budget impact assessment performed	17	49
Equity addressed at all in the paper	18	51
**Average Score**	**13**	**66**

### Quality of data sources

The data domains with data sourced from the pre-defined highest levels of evidence were epidemiology (34 studies; 80% high, 16% medium) and effectiveness (35 studies; 85% high, 4% medium). For epidemiological data, 46% of relevant parameters were based on data sourced from reliable databases specifically conducted for the study covering patients solely from the jurisdiction of interest. For instance, Manyema et al., (2016) identified Statistics South Africa as the primary source for population estimates by age and sex for 2012 [55] and Basu et al. (2016) used the World Health Organization Study on Global Ageing and adult health to determine population projections by age, sex and urban versus rural residence in each country [56]. In the case of effectiveness estimates, data were mostly drawn from meta-analyses and randomised controlled trials, although often from higher income settings. Notably the level of evidence in the data domains of resource use and costs were generally considered of high quality: resources and service use (35 studies; 74% high, 25% low) and costs (35 studies; 78% high, low 20%). Studies often sourced information from reliable databases that were within the same jurisdiction. These included the National Health Insurance Scheme and International Drug Price Indicator Guide in Nigeria [21]. The weakest data domains were outcome utilities (24 studies, 43% high) and equity (12 studies, 67% high).

**Table 4:** Data sources from 35 studies for the six domains of evidence ranked by six levels of hierarchies of evidence by the number of sources, ranked evidence level (high, medium, low), and proportion of all sources at that evidence level (%)

Data domain	Rank	Source (n)	Evidence Level	Level %
**Epidemiology**				
Case series or analysis of reliable administrative databases specifically conducted for the study covering patients solely from the jurisdiction of interest	**1**	9	High	80
Recent case series or analysis of reliable administrative databases covering patients solely from the jurisdiction of interest	**2**	32		
Recent case series or analysis of reliable administrative databases covering patients solely from another jurisdiction	**3**	8	Medium	16
Old case series or analysis of reliable administrative databases. Estimates from RCTs	**4**	0	Low	4
Estimates from previously published economic analyses: unsourced	**5**	1		
Expert opinion	**6**	1		
***Total (34 studies)***		***51***		
**Effectiveness**				
Meta-analysis of RCTs with direct comparison between comparator therapies, measuring final outcomes	**1 +** ^**a**^	30	High	85
Single RCT with direct comparison between comparator therapies, measuring final outcomes	**1**	18		
Meta-analysis of RCTs with direct comparison between comparator therapies, measuring surrogate* outcomes Meta-analysis of placebo-controlled RCTs with similar trial populations, measuring the final outcomes for each individual therapy	**2 +** ^**a**^	7		
Single RCT with direct comparison between comparator therapies, measuring the surrogate* outcomes Single placebo-controlled RCTs with similar trial populations, measuring the final outcomes for each individual therapy	**2**	12		
Meta-analysis of placebo-controlled RCTs with similar trial populations, measuring the surrogate* outcomes	**3 +** ^**a**^	3	Medium	4
Single placebo-controlled RCTs with similar trial populations, measuring the surrogate* outcomes for each individual therapy	**3**	0		
Case control or cohort studies	**4**	5	Low	11
Non-analytic studies, for example, case reports, case series	**5**	4		
Expert opinion	**6**	0		
***Total (35 studies)***		***79***		
**Resources & service use**				
Prospective data collection or analysis of reliable administrative data for specific study	**1**	9	High	74
Recently published results of prospective data collection or recent analysis of reliable administrative data – same jurisdiction	**2**	30		
Unsourced data from previous economic evaluations – same jurisdiction	**3**	1	Medium	2
Expert opinion	**4**	3	Low	25
Recently published results of prospective data collection or recent analysis of reliable administrative data – different jurisdiction	**5**	10		
Unsourced data from previous economic evaluation – different jurisdiction	**6**	0		
***Total (35 studies)***		***53***		
**Costs**				
Cost calculations based on reliable databases or data sources conducted for specific study – same jurisdiction	**1**	18	High	78
Recently published cost calculations based on reliable databases or data source – same jurisdiction	**2**	45		
Unsourced data from previous economic evaluation – same jurisdiction	**3**	2	Medium	2
Expert opinion	**4**	5	Low	20
Recently published cost calculations based on reliable databases or data sources – different jurisdiction	**5**	11		
Unsourced data from previous economic evaluation – different jurisdiction	**6**	0		
***Total (35 studies)***		***81***		
**Outcome (Utility)**				
Direct utility assessment for the specific study from a sample either: (a) of the general population; (b) with knowledge of the disease(s) of interest; (c) of patients with the disease(s) of interest Indirect utility assessment from specific study from patient sample with disease(s) of interest, using a tool validated for the patient population	**1**	8	High	43
Indirect utility assessment from a patient sample with disease(s) of interest, using a tool not validated for the patient population	**2**			
Direct utility assessment from a previous study from a sample either: (a) of the general population; (b) with knowledge of the disease(s) of interest; (c) of patients with the disease(s) of interest Indirect utility assessment from previous study from patient sample with disease(s) of interest, using a tool validated for the patient population	**3**	5	Medium	57
Unsourced utility data from previous study – method of elicitation unknown	**4**	17	Low	0
Patient preference values obtained from a visual analogue scale	**5**	0		
Delphi panels, expert opinion	**6**	0		
***Total (24 studies)***		***30***		
**Equity**				
Case series or analysis of reliable administrative databases specifically conducted for the study covering patients solely from the jurisdiction of interest	**1**	0	High	67
Recent case series or analysis of reliable administrative databases covering patients solely from the jurisdiction of interest	**2**	10		
Recent case series or analysis of reliable administrative databases covering patients solely from another jurisdiction	**3**	4	Medium	27
Old case series or analysis of reliable administrative databases. Estimates from RCTs	**4**	1	Low	7
Estimates from previously published economic analyses: unsourced	**5**	0		
Expert opinion	**6**	0		
***Total (12 studies)***		***15***		

a For ranking of effectiveness, there may be two levels within a rank to distinguish between evidence from a meta-analysis of trials (denoted by +) and a single trial

* Surrogate outcome is an endpoint measured in lieu of some other so-called true endpoint [16]

### Descriptive analysis of author comments on sources and quality

Three of four studies (n = 26, 74%) referred to data sources in the Discussion section of the publications, in many cases highlighting the challenges of obtaining good quality and locally relevant data (Additional File 1). Many of the ‘global’ or 'regional’ papers (e.g. from WHO) used regional estimates instead of country level estimates, for example [27, 29, 37]. There was often the need to use estimates from high income countries (HIC) and apply them to LMIC, particularly effectiveness estimates from trials. Authors highlighted concerns that there was an absence of rigorous data in the epidemiology domain especially with respect to risk factors, disease progression, prevalence of complications, and disease sequelae. Several authors noted that the perspective of the economic evaluation was important particularly for costs in the context of LMIC where there is high out of pocket expenditure [26, 35, 51]. Six studies made specific recommendations mostly covering empirical data collection [24, 26, 31, 32, 38, 53]. Only three studies said they explored data limitations in uncertainty analyses [36, 39, 43].

## Discussion

### Statement of principal findings

While authors highlighted concerns around the availability of good quality local evidence, most of the studies, particularly with respect to the domains of effectiveness and epidemiology, sourced data that were categorised as ‘high level’ in the domain-specific hierarchies of evidence applied [21, 24, 51]. Data sources within the domains of resource use and costs were generally obtained from reliable sources such as the relevant national statistics authority in the country of interest, or the International Drug Price Indicator Guide [21].

Although the reporting standards of the evaluations were high, adherence to methodological standards appeared uneven. For instance, three of five studies applied a 3% discount rate for both inputs and outputs as recommended by the iDSI Reference Case (some studies used more than one discount rate) [15]. While 3% has been adopted as a global health standard, there have been concerns that this does not reflect the economic context of LMICs, and over-values the future costs and benefits of interventions [57]. While most evaluations stated a time horizon, only 30% used a lifetime horizon as recommended by the iDSI Reference Case [15]. Typically, a lifetime horizon should be applied in economic evaluations (unless there are good reasons not to) and this is particularly relevant for NCDs given their chronic nature. It is usually appropriate to apply a time horizon that captures all relevant costs and outcomes pertaining to the decision problem.

Some studies used a Markov modelling approach to estimate benefits and costs over a longer period. Modelling approaches are more common in economic evaluations, especially for those involving the extrapolation of evidence beyond the duration of many trials [17]. Modelling analyses, compared to trial-based or empirical evaluations, generally apply longer time horizons, include more comparators, and are less restricted to generalisability issues in different settings or countries [17, 58, 59]. Furthermore, modelling approaches allow researchers to account for final patient-relevant endpoints, such as death or a cardiovascular event (e.g. stroke, myocardial infarction) rather than relying on surrogate or intermediate outcomes often measured in randomised controlled trials, such as a reduction in blood pressure or cholesterol levels [58]. We note, however, that modelling approaches are only as good as the assumptions on which they are based.

Many of the studies did not adequately explore uncertainty in their analyses. For example, only about half of the studies undertook probabilistic sensitivity analysis (PSA) despite recommendations that it should be routinely used to reflect the uncertainty in multiple parameters, and is especially important for evaluations characterised by non-linearities such as Markov models where PSA provides the best estimates of the mean costs and benefits [60]. Typically, Markov models are used in the evaluation of NCD interventions [61] and as such, the expectation is that PSA would be applied in every instance unless there are good reasons not to.

For most of the cost-effectiveness studies included, DALYs were used as the main outcome measure. Some studies included both an outcome in natural health units and a generic preference-based measure. The appropriateness or otherwise of the use of DALYs in the evaluation of NCD interventions has been debated elsewhere [62]. Nevertheless, DALYs are the most used metric in LMIC settings due to the lack of locally relevant data required to translate outcomes from clinical trials and other studies for the calculation of QALYs [15]. Increasing interest in the use of QALYs for HTA assessment in Africa has recently led to several health state valuation studies in Ethiopia and Uganda - a necessary precursor for the more widespread use of this metric [63–65].

The published economic evaluations we reviewed were relatively weak with respect to the quality of their reporting and methodological adherence to the iDSI Reference Case in the areas of budget impact analysis and equity. This is broadly consistent with the findings of an earlier review of cost-per DALY averted studies and their adherence to the iDSI Reference Case, which found very low levels of consideration of these aspects by the authors of included studies [19].

### Strengths and weaknesses of this study

This is the first study, to our knowledge, to examine the quality of data sources across six domains in economic evaluations in SSA focused on the NCDs using a comprehensive and systematic search combined with reputable scoring systems across four aspects of economic evaluations. This study outlines as a novel approach to assessing the quality of data sources with a combination of a methods/reporting checklist (based on the iDSI Reference Case [19]) with six data domain-specific hierarchies derived from earlier studies [16].

There were some limitations with our study. Firstly, we will likely have missed studies not published in journals that were indexed in the selected databases or present in the grey literature. This may have reduced the yield as some economic evaluations conducted in LMICs are not published due to uncontrollable factors such as economic constraints [66]. Furthermore, evaluations from LMICs which are published generally reflect those of higher quality so we may have overestimated the quality of the data sources used. Secondly, the method of scoring the quality of the data sources had the potential to be skewed by one or two very good quality studies, particularly those with more sources of data. Generally, the economic evaluations which scored poorly would use only single or limited data sources for each data domain. There are other checklists for economic evaluations, such as CHEERS and Drummond [67, 68]; we reviewed these checklists and ascertained that the important elements were captured in the general characteristics and iDSI reference case standards [15].

There remains an issue regarding the applicability of evidence in a given jurisdiction when sourced from another setting, even when that evidence is judged as high quality, such as a meta-analysis of randomised controlled trials. Authors in the reviewed studies noted concerns regarding the use, for example, of trial evidence from high income settings. In our analysis, we did not explore the broader issue of evidence transferability, although we recognise this is also arguably a component of any ‘quality’ assessment. Checklists to support evidence transfer for the purpose of HTA are available, although it has been argued that more guidance may be warranted in settings with limited capacity to undertake HTA [69]. Although not specifically analysed in this study, we anecdotally noted that the overall quality of the economic evaluations improved over the twenty-year time frame. The studies ranged from rudimentary in a single setting to sophisticated and extensive economic evaluations across many countries and regions (often with many data sources). We speculate that important publications and guidance (e.g. WHO-CHOICE and the publication of guides for HTA methods including the iDSI Reference Case [15]) may have contributed to the evolution of better quality evaluations.

### Strengths and weaknesses relating to other studies

The findings of this review are consistent with those found in other studies focused on LMICs. Teerawattananon et al., reported that data sources used for costs were from high quality sources (prospectively collected from reliable and local databases), and the evidence used for clinical effectiveness was of lower quality primarily obtained from single placebo-controlled clinical trials in another jurisdiction [70]. Furthermore, Prinja et al. (2015) encountered similar issues regarding the availability of evidence in India; there was a lack of locally available evidence on disability or quality of life weights, hence most cost utility analyses used utility weights from non-Indian settings [71]. In terms of the reporting and methodological results, there was a common trend seen amongst the studies. In India, it was found that the areas which required the most improvement was the perspective, justification on the type of economic evaluation used, discount rates, costing methodologies, and approaches to exploring uncertainty, especially in model-based evaluations [71]. A major weakness in the methodological and reporting standards for studies from Thailand was the lack of an incremental cost effectiveness ratio and the limited use of uncertainty (sensitivity) analyses. Overall, our results are very similar to what others have found in LMIC.

### Implications for practice

Many of the problems we identified in relation to the quality of the data sources used and the reporting and methodological standards stem from a lack of robust and comprehensive local data. The lack of good quality data ultimately limits countries in SSA from generating quality evidence to support decision making. By using data of poorer quality, policy makers risk making decisions that are not suited to their local context. This becomes particularly problematic when a decision is made to fund one health intervention over another given the opportunity costs involved [15].

This study highlights the importance for SSA countries to establish effective data governance frameworks to improve the production, processing, protection, ownership, quality, openness, timeliness, relevance, accessibility, and interoperability of different types of data [72]. This can be achieved through significant investments in data technologies, platforms, and tools such as internet and mobile digital technologies. LMICs are starting to implement such systems. For instance, India has analysed the cost-effectiveness of utilising a cloud-based emergency health care information system through the use of palm vein pattern recognition [73]. It avoided misinterpretations of data amongst collectors and participants and reduced errors, physical storage issues, and security and privacy concerns [73]. The use of mobile phone-based applications to collect data in the primary care context in Ethiopia significantly improved the quality, timeliness, and processing of data [74]. Biometric fingerprint scanning in Bangladesh reduced the gap in identification by advancing the civil registration and vital statistics systems thereby enhancing epidemiological data and the monitoring of service delivery [75].

Furthermore, there needs to be greater collaboration and coordination among data collectors (government, private-sector, and civil society) to reduce duplication of results by increasing the availability and accessibility of data [72]. Both policymakers and researchers (i.e. those generating economic evaluations) need to commit to improving the production and use of data. This can be achieved by building the research capacity of those conducting economic evaluations; and creating an enabling environment for more research efforts that are locally relevant and of good quality [17]. Currently, researchers are hindered in producing high-quality research due to budget restrictions and policymakers are reluctant to use evidence derived from poorer-quality data [17]. Policymakers are encouraged to acknowledge the contribution which economic evaluations can make towards better priority setting and resource allocation.

Ongoing efforts are needed to address the political and economic issues surrounding data; there has been suboptimal engagement with policymakers to appreciate the importance of data [76]. While this study only identified whether studies declared a conflict of interest and the funding source, Glassman et al. (2012) emphasises the detrimental impact that international donors have on the quality of data in LMIC, especially when there are incentives present [9]. Misinterpretations and systematic bias have been found, where countries are reporting slower rates of growth and poverty reduction to maintain international financing [9]. This significantly impacts the quality of data produced, and future efforts will need to minimise the political interference.

### Future research

As economic evaluations seek to provide evidential and analytical support for decision making, more funding for conducting research, developing technical capacity, and creating evidence related specifically to the SSA setting is required [77]. An ideal starting point would be growing data communities and investing in the primary data collectors. There are two main priorities for future research. Firstly, a major area is to review the training and level of investment of SSA countries to build the technical capacity of their researchers conducting economic evaluations; it will improve the reporting and methodological adherence. Secondly, given the dearth of locally relevant data on clinical effectiveness, we could enhance the transferability of data across jurisdictions and explore techniques combining randomised and non-randomised (‘real world’ evidence) [78]. The adaptation of evidence and evaluations from high income countries to LMICs is a possibility, but will be challenging and need resources [79]. Some studies have reviewed the existing approaches for assessing the geographic transferability of data sources [80, 81]. Goeree et al., (2011) reviewed seven unique systems for assessing transferability where there was high variability among the proposed approaches. Overall, due to the complexities in identifying appropriate weights, it still remains uncertain as to whether data sources were appropriate to be transferred [80]. We note ongoing research on the quality of reporting and data sources used in economic evaluations [82].

## Conclusion

We examined the quality of data sources used in published economic evaluations in SSA in the areas of CVD and diabetes using a novel approach to rank and describe data quality. The data sources in some domains are considered high quality but there remains a need to make substantial improvements in the methodological adherence and overall quality of data sources to provide evidence that is sufficiently robust to support decision making in SSA within the context of UHC and health benefits plans. Many SSA governments will need to strengthen and build their capacity to conduct economic evaluations of interventions and health technology assessment for improved priority setting. This capacity building includes enhancing local infrastructures for routine data production and management. If many of the policy makers are using economic evaluations to guide resource allocation, it is imperative that the evidence used is of the feasibly highest quality.

### Electronic supplementary material

Below is the link to the electronic supplementary material.

Supplementary Material 1

## Data Availability

All data necessary for interpretation of this study are contained in the manuscript and additional files.

## Abbreviations

CEA: cost effectiveness analysis
CUA: cost utility analysis
CVD: cardiovascular disease
DALY: disability adjusted life year
HTA: health technology assessment
iDSI: International Decision Support Initiative
LMIC: low and middle income countries
NCD: non-communicable diseases
PSA: probabilistic sensitivity analysis
QALY: quality adjusted life year
SDG: sustainable development goals
SSA: Sub-Saharan Africa
UHC: universal health coverage
WHO: World Health Organization
